# Down-regulation of β-arrestin2 promotes tumour invasion and indicates poor prognosis of hepatocellular carcinoma

**DOI:** 10.1038/srep35609

**Published:** 2016-10-19

**Authors:** Wu-Yi Sun, Shan-Shan Hu, Jing-Jing Wu, Qiong Huang, Yang Ma, Qing-Tong Wang, Jing-Yu Chen, Wei Wei

**Affiliations:** 1 Institute of Clinical Pharmacology of Anhui Medical University, Hefei 230032, China; 2 Key Laboratory of Anti-inflammatory and Immune Medicine, Ministry of Education, Hefei 230032, China; 3 Anhui Collaborative Innovation Center of Anti-Inflammatory and Immune Medicine, Hefei, 230032, China

## Abstract

β-arrestins, including β-arrestin1 and β-arrestin2, are multifunctional adaptor proteins. β-arrestins have recently been found to play new roles in regulating intracellular signalling networks associated with malignant cell functions. Altered β-arrestin expression has been reported in many cancers, but its role in hepatocellular carcinoma (HCC) is not clear. We therefore examined the roles of β-arrestins in HCC using an animal model of progressive HCC, HCC patient samples and HCC cell lines with stepwise metastatic potential. We demonstrated that β-arrestin2 level, but not β-arrestin1 level, decreased in conjunction with liver tumourigenesis in a mouse diethylnitrosamine-induced liver tumour model. Furthermore, β-arrestin2 expression was reduced in HCC tissues compared with noncancerous tissues in HCC patients. β-arrestin2 down-regulation in HCC was significantly associated with poor patient prognoses and aggressive pathologic features. In addition, our *in vitro* study showed that β-arrestin2 overexpression significantly reduced cell migration and invasion in cultured HCC cells. Furthermore, β-arrestin2 overexpression up-regulated E-cadherin expression and inhibited vimentin expression and Akt activation. These results suggest that β-arrestin2 down-regulation increases HCC cell migration and invasion ability. Low β-arrestin2 expression may be indicative of a poor prognosis or early cancer recurrence in patients who have undergone surgery for HCC.

Hepatocellular carcinoma (HCC) is the fifth most common type of malignancy and the third leading cause of cancer-related death worldwide^1^. The treatment options for HCC are limited. Surgical resection and liver transplantation are the only curative treatments, but most patients are ineligible for surgery because they are often diagnosed at late stages of the disease^2^,^3^. Despite advances in HCC diagnosis and treatment in recent decades, the prognoses of patients with advanced HCC remain very poor, mainly due to the high rates of recurrence and metastasis associated with the disease^4^. Therefore, it is necessary to elucidate the molecular pathogenesis of HCC to identify novel markers with which to diagnose and treat this deadly disease, as well as to determine its prognosis in affected patients^5^.

The arrestin family comprises the following four members: β-arrestin1, β-arrestin2, x-arrestin, and s-arrestin^6^. β-arrestin1 and β-arrestin2 have been extensively studied and are ubiquitously expressed, whereas x-arrestin and s-arrestin are found exclusively in the visual system. Both β-arrestin1 and β-arrestin2 can transduce G protein-coupled receptor (GPCR) signals by forming protein complexes with signalling molecules downstream of G protein to mediate GPCR desensitization, internalization, degradation and recycling^7^,^8^. β-arrestins have recently been found to play new roles in regulating intracellular signalling networks associated with malignant cell functions, including the extracellular regulated kinase (ERK), c-Jun NH_2_-terminal kinase (JNK), and phosphoinositide 3-kinase (PI3K)-Akt^9^,^10^. Altered β-arrestins expression levels have been reported in many cancers, including lung cancer^11^, colorectal cancer^12^, ovarian cancer^13^, bladder cancer^14^, and breast cancer^15^.

Although β-arrestins can transduce multiple signals in cells, little is known about their participation in HCC progression. To date, no studies have reported the clinicopathologic significance of β-arrestins in HCC. This study was designed to investigate the role of β-arrestins in HCC and HCC cell invasion. Our current studies of mouse diethylnitrosamine (DEN)-induced liver tumour models demonstrate that gradual decreases in β-arrestin2 expression, but not β-arrestin1 expression, are associated with hepatocarcinogenesis. To assess the importance of β-arrestins in HCC, tumour samples from HCC patients were analysed. We found that β-arrestin2 is expressed in low levels in HCC tissues compared with peritumoural tissues, and that its low expression is strongly associated with aggressive pathologic features and is predictive of a poor prognosis in HCC patients after surgery. Furthermore, β-arrestin1 and β-arrestin2 expression in human HCC cell lines with stepwise metastatic potential was evaluated *in vitro*. The data presented herein indicate that β-arrestin2 expression gradually decreases with increasing HCC cell line metastatic potential, and that β-arrestin2 overexpression inhibits HCC cell metastasis and invasion, reduces Akt activation, increases E-cadherin expression, and decreases vimentin expression. Altogether, these findings suggest that β-arrestin2 acts by down-regulating the Akt pathway to inhibit HCC cell metastasis and invasion. β-arrestin2 may therefore has potential as a prognostic and treatment marker in HCC.

## Results

### Decreases in β-arrestin2 expression in liver tumourigenesis *in vivo*

**Figure 1:**
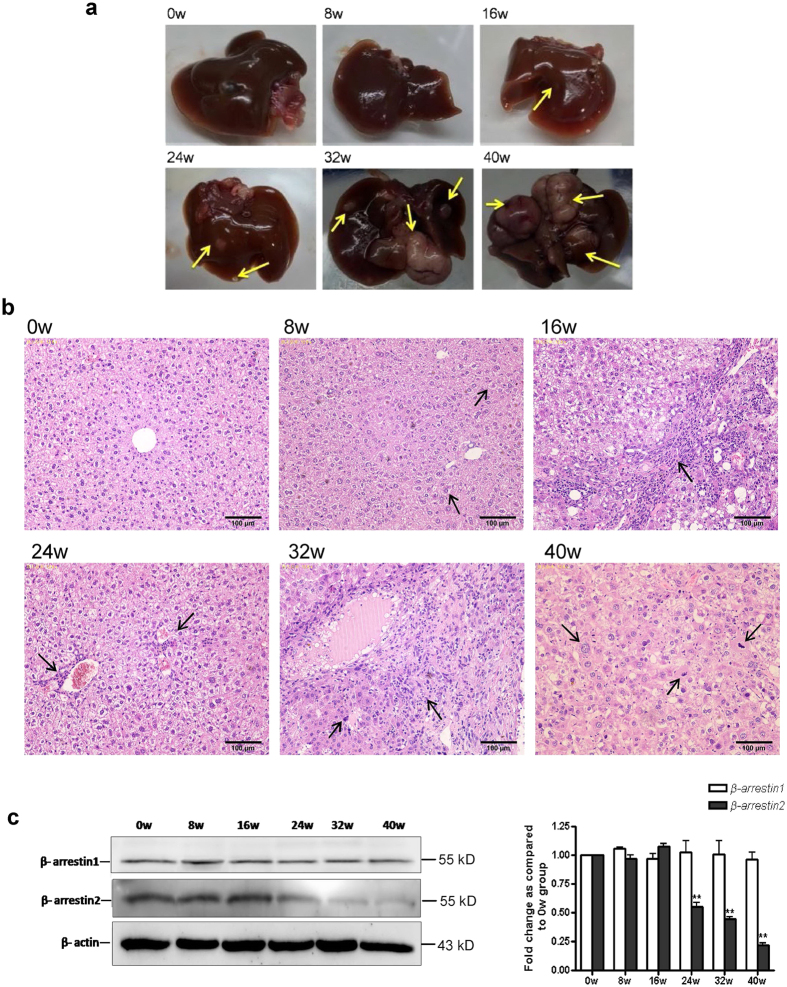
Time course analysis of the liver at different time points in DEN-induced liver tumourigenesis. (**a**) Representative photographs of liver tissue samples from control mice and from mice that developed tumours post-DEN injection. (**b**) Representative photographs of H&E staining of tissue samples from mice treated with DEN at different time points. (**c**) Time course analysis of β-arrestin1 and β-arrestin2 expression by Western blotting in DEN-treated mice. The protein quantification bar graph was plotted from no less than 3 independent experiments. The densitometry values in the histograms are expressed as fold changes relative to week 0, which was assigned a value of 1. The data from three independent experiments are shown as the mean ± SD. **P < 0.01 compared with the week 0 group.

To study the dynamic expression of β-arrestin1 and β-arrestin2 *in vivo*, we established a mouse DEN-induced liver tumour model to investigate the changes in the expression of these proteins during HCC progression. Significant increases in liver size were observed as early as 8 weeks after DEN injection, and these increases continued throughout the study period. Additionally, colour changes were noted in DEN-treated livers, as they became more inhomogeneous, spotty and paler than normal control livers. No hepatic nodules were visible in the livers of normal mice. Very few small nodular lesions were noted in the liver parenchyma at 16 weeks after DEN injection. However, prominent macroscopic nodules were noted in DEN-treated mice at 32 weeks after injection. These nodules ultimately occupied most of liver surface (Fig. 1a).

Haematoxylin and eosin (H&E) staining of the livers of normal mice demonstrated anastomosing plates of hepatocytes radiating from the centrilobular venule towards the periphery of the hepatic lobule, as shown in Fig. 1b. These hepatocytes were tightly packed and exhibited pink staining and round violet nuclei containing prominent nucleoli. The liver sections exhibited intra-lobular inflammatory cell infiltration, and various degrees of fibrosis, hepatocyte injury and degeneration at 16 weeks post-DEN injection. The liver sections exhibited significant hepatocyte architecture loss at 32 weeks post-injection, as demonstrated by the presence of oval- or irregular-shaped cells. Binucleated pleomorphic and hyperchromatic large cells characterized by a central nucleolus and an enlarged cytoplasm could be clearly distinguished from adjacent normal parenchymal cells. Additionally, extensive vacuolation was noticed in the cytoplasm with masses of acidophilic material.

Western blot analysis was used to investigate β-arrestins expression in liver tumourigenesis. The results showed that β-arrestin2 protein levels decreased significantly in mouse liver tissues (24, 32, and 40 weeks) in conjunction with liver tumourigenesis. Furthermore, DEN-treated mice exhibited significantly lower β-arrestin2 expression than normal control mice (Fig. 1c). However, there was no difference in β-arrestin1 expression between DEN-treated mice and normal control mice. We also observed that β-arrestin2 expression did not change significantly with age in normal C57BL/6J mice (data not shown), indicating that ageing does not induce decreases in β-arrestin2 expression. Altogether, our results suggest that greater mouse liver tumourigenesis is significantly associated with decreased β-arrestin2 expression.

### β-arrestin2 was frequently down-regulated in human HCCs

**Figure 2:**
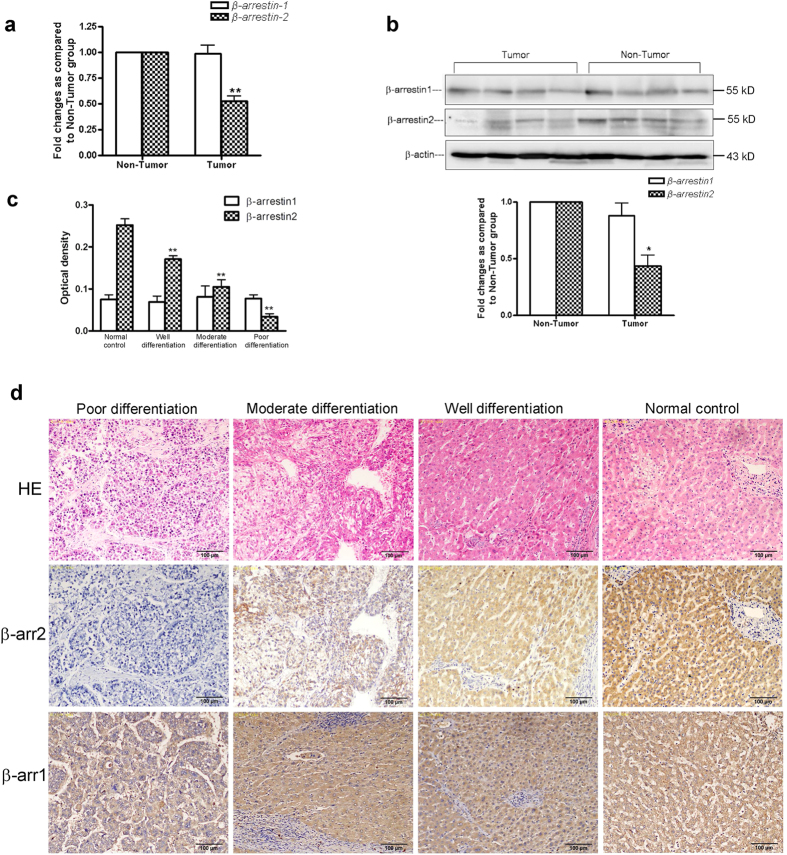
β-arrestin1 and β-arrestin2 expression in liver tissues. (**a**) Transcriptional β-arrestin1 and β-arrestin2 expression in tumour tissues and non-tumour tissues. (**b**) Representative Western blot analysis results for β-arrestin1 and β-arrestin2 expression in HCC and noncancerous liver tissue lysates. The densitometryvalues in the histograms are expressed as fold changes relative to non-tumour group, which was assigned a value of 1.The data from three independent experiments are shown as the mean ± SD. ^*^P < 0.05 *vs* non-tumour group. (**c**) Positive optical density values of β-arrestin1 and β-arrestin2 expres.sion. ^**^P < 0.01 compared with the normal control group. (**d**) Immunohistochemical analysis of β-arrestin1 and β-arrestin2 expression in normal liver tissues and HCC tissues with different histological tumour grades. The top panel shows H&E staining of serial sections. Immunohistochemistry demonstrated relatively high β-arrestin2 expression in well-differentiated HCC cells, moderate expression in moderately differentiated HCC cells, and only faint or negative expression in poorly differentiated HCC cells (Original magnification ×200).

To verify the roles of β-arrestins in HCC further, we first examined the profiles of β-arrestin1 and β-arrestin2 expression in 19 pairs of tumour and adjacent noncancerous liver tissue samples via quantitative real-time reverse transcription polymerase chain reaction (qRT-PCR) and Western blot analysis. β-arrestin1 and β-arrestin2 expression bands varied across these samples. qRT-PCR demonstrated that β-arrestin2 mRNA was down-regulated in HCC tissues by at least 48% compared with noncancerous liver tissues (Fig. 2a). However, no significant β-arrestin1 mRNA down-regulation was observed in HCC tissues. Western blot analysis confirmed that β-arrestin2 expression was significantly lower in HCC tissues than in noncancerous liver tissues (Fig. 2b). This finding is consistent with the abovementioned decreases in β-arrestin2 mRNA expression that occur in HCC tissues. Similar levels of β-arrestin1 protein expression were observed in all materials examined (Fig. 2b).

### β-arrestin2 down-regulation was associated with aggressive tumour behaviour and poor patient survival

We next investigated the association between β-arrestin2 dysregulation and disease progression via immunohistochemical staining of paraffin embedded primary HCC tumour samples with different histological grades (well-differentiated, moderately differentiated, or poorly differentiated) from 75 HCC patients and 18 histologically normal controls. Different HCC histological grades demonstrated different β-arrestin2 immunoreactivity (Fig. 2d). Well-differentiated HCC exhibited higher β-arrestin2 expression than the other HCC histological grades, as β-arrestin2 expression decreased from well-differentiated to poorly differentiated HCC. Furthermore, β-arrestin2 expression levels were significantly lower in HCC livers than in normal control livers (*P* < 0.01). Positive β-arrestin1 staining was also observed in liver tissues. There was no significant difference in β-arrestin1 expression among well-differentiated, moderately differentiated and poorly differentiated HCC tissues and normal liver tissues (Fig. 2c). HCC patient β-arrestin2 expression scores are summarized in Table 1. Clinicopathologic analysis revealed that β-arrestin2 down-regulation in HCC was significantly associated with aggressive pathologic features, including advanced tumour stage (P = 0.003), metastasis (P = 0.024), poor tumour cell differentiation (P = 0.040) and large tumour size (P = 0.012).

**Figure 3:**
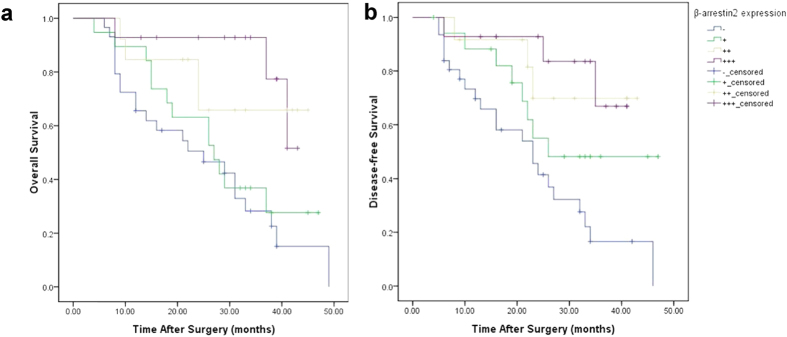
Kaplan-Meier analysis of overall survival (**a**) and disease-free survival (**b**) in 75 patients based on β-arrestin2 expression. High β-arrestin2 expression was associated with longer survival and disease-free survival than low β-arrestin2 expression.

Furthermore, to determine the prognostic significance of β-arrestin2 down-regulation in HCC, we analysed the correlation between β-arrestin2 expression in HCC and patient survival. We found that low β-arrestin2 expression was associated significantly with poorer 3-year overall survival rate (P = 0.004) and 3-year disease-free survival rate (P = 0.002) in our HCC cohort (Fig. 3). Collectively, our findings suggest that loss of β-arrestin2 expression may play an important role in HCC metastasis.

### Low β-arrestin2 expression was significantly correlated with HCC metastasis

**Figure 4:**
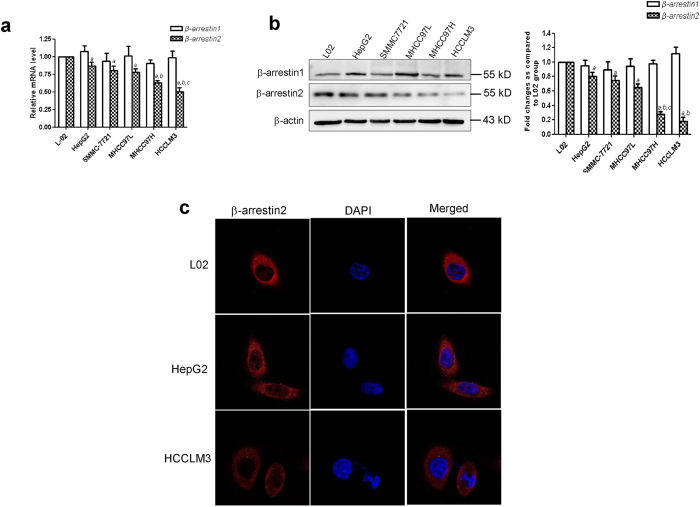
β-arrestin1 and β-arrestin2 expression in HCC cell lines with stepwise metastasis potential. (**a**) qRT-PCR analysis of relative β-arrestin1 and β-arrestin2 mRNA levels in different cell lines. (**b**) Western blot analysis of β-arrestin1 and β-arrestin2 protein expression in the immortalized normal liver cell line, L02; the HCC cell lines, HepG2, SMMC-7721 and MHCC97L, MHCC97H, HCCLM3. The densitometry values in the histograms are expressed as fold changes relative to the L-02 group, which was assigned a value of 1. The data from three independent experiments are shown as the mean ± SD (a: *P* < 0.05 compared with L-02; b: *P* < 0.05 compared with HepG2, SMMC-7721; c: *P* < 0.05 compared with MHCC97L). (**c**) β-arrestin2 subcellular localization examined by immunofluorescence confocal microscopy. Nuclei were stained with DAPI.

According to our qRT-PCR results, β-arrestin2 expression levels were negatively correlated with HCC cell line metastatic potential. HCCLM3, the HCC cell line with the highest metastatic potential, exhibited significantly lower β-arrestin2 expression than the other HCC cell lines and the L-02 cell line (Fig. 4a). Consistent with these qRT-PCR data, we observed that β-arrestin2 protein was expressed at a higher level in immortalized normal L-02 liver cells than in HCC cells. β-arrestin2 exhibited reduced expression in HepG2 and SMMC-7721 cells compared with L-02 cells and exhibited minimal expression in highly metastatic MHCC97H and HCCLM3 cells (Fig. 4b). β-arrestin2 expression and subcellular localization was confirmed via immunofluorescence confocal microscopy (Fig. 4c). Immunofluorescence analysis showed that β-arrestin2 was expressed predominantly in the cytoplasm but was also partially expressed in the cytoplasmic membrane in L-02, HepG2 and HCCLM3 cells. These immunofluorescence staining intensity results were in accordance with our qRT-PCR and Western blotting results, suggesting that β-arrestin2 expression gradually decreased in conjunction with HCC development.

### β-arrestin2 overexpression suppressed HCC cell migration and invasion *in vitro*

**Figure 5:**
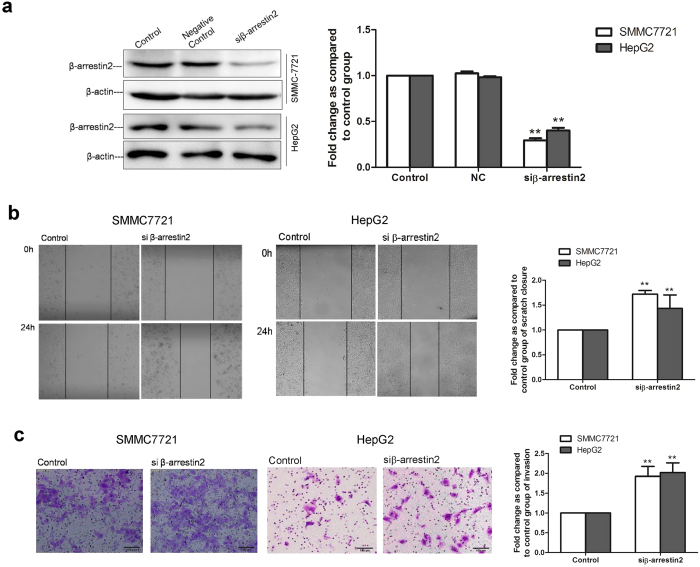
β-arrestin2 knockdown by siRNA increases HCC cell migration and invasion. (**a**) β-arrestin2 knockdown in SMMC-7721 cells and HepG2 cells, as confirmed by Western blot analysis. β-arrestin2 band intensity was quantified by densitometry and normalized to β-actin. The densitometry values in the histogram are expressed as fold changes relative to the control group, which was assigned a value of 1. The data from three independent experiments are shown as the mean ± SD. ^**^P < 0.01 compared with the control group. (**b**) Representative photographs and a bar graph of the wound-healing assay are shown. The black line was used to mark the ranges of the scratches. (**c**) Transwell Matrigel invasion assays showed that the number of invasive cells in the β-arrestin2 siRNA-treated group increased significantly compared with the number of invasive cells in the control group. ^**^P < 0.01 compared with the control group.

To determine whether β-arrestin2 is involved in HCC cell migration and invasion, we used small interfering RNA (siRNA) targeting β-arrestin2 mRNA to determine the effects of endogenous β-arrestin2 expression on HCC cell migration and invasion. We transfected siRNA targeting β-arrestin2 or scrambled RNA into SMMC-7721 cells and HepG2 cells exhibiting relatively higher endogenous β-arrestin2 expression. β-arrestin2 protein expression was significantly reduced, as determined by Western blot analysis (Fig. 5a). Subsequent experiments showed that when the β-arrestin2 expression was reduced by β-arrestin2 siRNA, SMMC-7721 and HepG2 cell migration and invasion ability increased significantly compared with control cell migration and invasion ability (Fig. 5b,c). These results indicate that β-arrestin2 down-regulation is associated with HCC cell migration and invasion.

**Figure 6:**
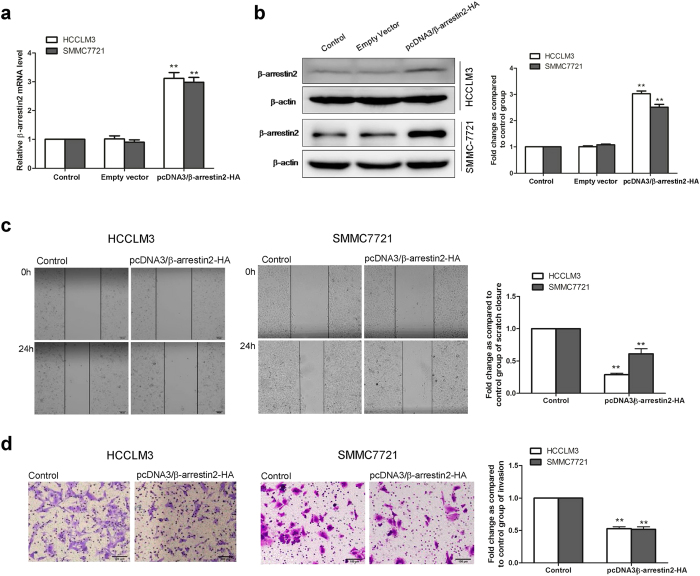
β-arrestin2 overexpression suppressed HCC cell migration and invasion. β-arrestin2 was overexpressed in the HCCLM3 and SMMC-7721 cell lines via HA-β-arrestin2-encoding plasmid transfection. Successful β-arrestin2 overexpression was confirmed by qRT-PCR (**a**) and Western blotting (**b**). β-arrestin2 expression values were calculated as fold changes relative to the control, which was assigned a value of 1. (**c**) β-arrestin2 overexpression significantly impeded HCCLM3 and SMMC-7721 cell migration ability. (**d**) Transwell Matrigel invasion assays showed that the number of invasive cells in the β-arrestin2-overexpression group decreased significantly compared with the number of invasive cells in the control group. ^**^P < 0.01 compared with the control group.

As we observed that loss of β-arrestin2 was closely associated with human HCC aggressiveness and metastases, we postulated that β-arrestin2 overexpression in HCC cells impedes HCC cell migration and invasion ability. Thus, we transfected plasmids encoding HA-β-arrestin2 into HCCLM3 cells and SMMC-7721 cells. Successful β-arrestin2 overexpression was confirmed by qRT-PCR (Fig. 6a) and Western blotting (Fig. 6b). We also performed a wound-healing assay of *in vitro* cell migration, in which a ~300 μm-wide linear strip of cells was scraped from a confluent monolayer using a pipette tip. Wound closure was quantified from serial micrographs, as shown in Fig. 6c. The wound-healing assay showed that wound closure was significantly decreased in β-arrestin2-transfected cells compared with control tumour cells at 24 h after wound infliction (Fig. 6c). *In vitro* tumour cell invasion analysis was performed via transwell invasion assay. Figure 6d shows that β-arrestin2 overexpression suppressed HCC cell invasion ability, as demonstrated by decreases in migrated cells. Our results indicate that β-arrestin2 suppresses HCC metastasis by negatively regulating HCC cell migration and invasion ability.

### β-arrestin2 inhibited HCC cell migration and invasion through Akt pathway down-regulation

**Figure 7:**
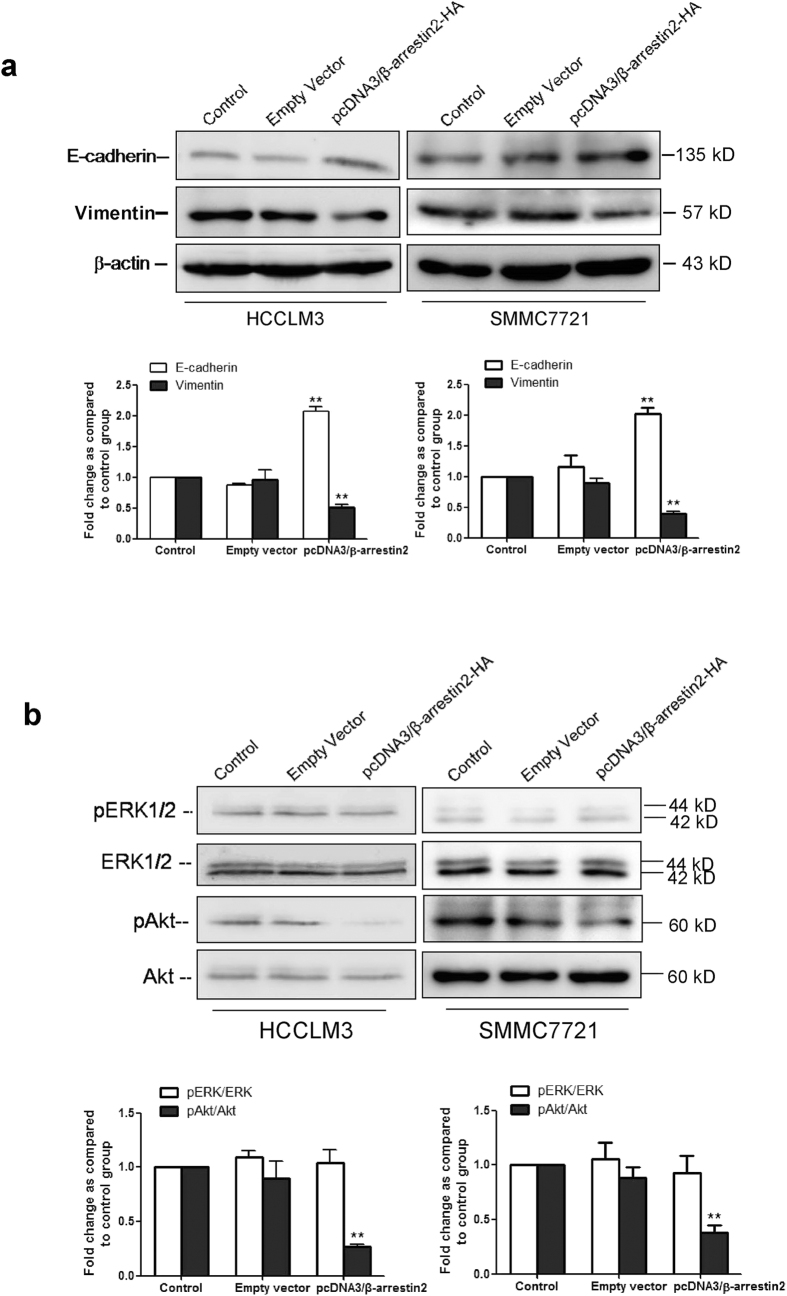
Effects of β-arrestin2 overexpression on E-cadherin and vimentin expression and the ERK 1/2 and Akt signalling pathways. (**a**) The EMT biomarkers E-cadherin and vimentin were detected by Western blotting. E-cadherin expression increases and vimentin expression decreases after pcDNA3/β-arrestin2-HA transfection are shown. (**b**) β-arrestin2 overexpression inhibited Akt activation but had no effect on ERK 1/2 phosphorylation. Columns represent the ratios of phospho-ERK1/2 to total ERK1/2 and phospho-Akt to total Akt. Data were normalized to the ratio of the control, which was assigned a value of 1 in the graphical presentation. In immunoblotting assay, gels were run under the same experimental conditions. ^**^P < 0.01 compared with the control group.

To investigate the effects of β-arrestin2 on epithelial-to-mesenchymal transition (EMT), we assessed the expression of two EMT markers: E-cadherin and vimentin^16^. E-cadherin was up-regulated, and vimentin was down-regulated in conjunction with β-arrestin2 overexpression in HCCLM3 cells and SMMC-7721 cells, as demonstrated by Western blot analysis (Fig. 7a).

Previous studies have shown that the ERK/MAPK and Akt signalling pathways are important for cancer development and cancer cell invasion^17^,^18^,^19^,^20^. It has also been reported that β-arrestin2 plays a critical role in MAPK and Akt pathway regulation^21^,^22^. We therefore examined whether these proteins participate in β-arrestin2-mediated inhibition of HCC cell migration and invasion. We introduced expression vectors carrying β-arrestin2 into HCCLM3 cells and SMMC-7721 cells via transfection. Forty-eight hours after transfection, we examined ERK1/2 and Akt expression and phosphorylation. Decreased Akt phosphorylation (pAkt) was observed in β-arrestin2-overexpressing cells compared with empty vector-transfected cells. By contrast, ERK phosphorylation was detected in all cells overexpressing β-arrestin2 and cells transfected with the empty vector (Fig. 7b). These results suggest that β-arrestin2 inhibits HCC cell migration and invasion via Akt signalling pathway inhibition.

## Discussion

HCC is one of the most common fatal malignancies worldwide^1^. The high mortality rate associated with HCC is related mainly to its presentation at advanced disease stages, as well as its frequent metastasis and recurrence after surgical resection. Tumour invasion and metastasis is the major cause of HCC tumour recurrence, but promising HCC metastasis therapies are not available^3^. Hence, identifying the factors associated with tumour metastasis and the molecular mechanisms underlying tumour progression is very important^23^.

β-arrestin1 and β-arrestin2, the main members of the arrestin family, are ubiquitously expressed. β-arrestins are classically known to regulate GPCR signalling through receptor desensitization and internalization^24^. Many recent studies have demonstrated that β-arrestins unexpectedly function as scaffold proteins for many signalling molecules in the cytoplasm and nucleus, thus regulating gene expression and cellular responses^25^. Mounting evidence indicates that aberrant β-arrestins expression are involved in several types of tumours. β-arrestins have been implicated in cell survival^26^, apoptosis^27^, migration^15^, and tumour growth^11^, but their clinical relevance in terms of HCC progression and metastasis has never been elucidated. Our studies are designed to examine the role of β-arrestin2 in hepatocarcinogenesis. The data presented in this work demonstrate that β-arrestin2 plays a crucial role in tumour growth, invasion, and metastasis. First, we demonstrated that β-arrestin2 expression was decreased in mouse DEN-induced liver tumour tissues compared with normal control tissues via Western blotting analysis. Second, we showed that β-arrestin2 expression was decreased in HCC patient tissues compared with noncancerous liver tissues via qRT-PCR, Western blotting and immunohistochemical staining analyses and demonstrated that its low expression is significantly associated with aggressive pathologic features and is predictive of poor HCC patient prognoses after surgery. Third, we confirmed that β-arrestin2 expression gradually decreases in conjunction with increases in HCC cell line metastatic potential. Finally, the results of our *in vitro* depletion and overexpression experiments indicate that β-arrestin2 can inhibit HCC cell metastasis and invasion by down-regulating Akt activation and vimentin expression and up-regulating E-cadherin expression.

Several preclinical models have been used to elucidate the molecular and cellular bases of HCC in patients, including rodent DEN treatment models. DEN is a carcinogen commonly used to induce HCC in rodent models. A single dose of DEN causes DNA damage and acute hepatitis development in 2-week-old mice, which ultimately leads to HCC development in these mice at approximately 8–10 months of age. DEN induces DNA adducts in hepatocytes undergoing cell division, eventually leading to HCC development^28^. The DEN rodent model has several advantages, as it induces HCC at a high rate and is highly reproducible^29^. Furthermore, DEN treatment facilitates the study of important molecular and cellular pathways involved in HCC development and is a valuable tool for investigating the ability of particular molecules to inhibit or promote liver cancer formation^30^,^31^,^32^. We used the DEN-induced liver tumour model in this study, employing Western blotting to detect dynamic β-arrestin1 and β-arrestin2 expression in mouse liver tissue samples. Our results indicated that β-arrestin2 protein expression was lower in mice liver tumour tissues than in normal liver tissues.

Some studies have shown that aberrant β-arrestin2 protein expression is associated with various types of human malignancies^33^. For instance, β-arrestin2 is highly expressed in the highly metastatic MDA-MB-231 breast cancer cell line and breast cancer tumours, and β-arrestin2 mediates human breast cancer cell migration and invasion^15^. A recent investigation revealed that β-arrestin2 is essential for the initiation and growth of intestinal tumours exhibiting elevated Wnt pathway activity^34^. By contrast, an increasing number of studies have indicated that β-arrestin2 expression is decreased in tumours. β-arrestin2 depletion promoted tumour growth and angiogenesis in a murine model of lung cancer^11^. Moreover, serum β-arrestin2 levels were significantly lower in non-small cell lung cancer (NSCLC) patients than in healthy controls, and NSCLC patients with high serum β-arrestin2 levels had better prognoses than patients with lower levels^35^. In addition, β-arrestin2 acts as a corepressor of androgen receptor (AR) signalling in prostate cancer, and AR expression and activity was negatively correlated with β-arrestin2 expression in human prostate tissues^36^. However, no clinical studies regarding the association between β-arrestin2 expression and HCC tumour metastasis and progression have been reported. In this study, we investigated the association between β-arrestin2 expression and HCC clinicopathologic characteristics. We found that β-arrestin2 expression was down-regulated in advanced HCC and that β-arrestin2 down-regulation was significantly correlated with HCC tumour invasive features, including advanced tumour stage, metastasis, poorer tumour cellular differentiation and larger tumour size. In addition, decreased β-arrestin2 expression in HCC tissues was significantly associated with poor overall survival in HCC patients. The results suggest that loss of β-arrestin2 expression may play an important role in HCC metastasis. Our *in vitro* studies support these findings and provide additional evidence that β-arrestin2 expression gradually decreases in conjunction with increases in HCC cell metastatic potential. Thus, low β-arrestin2 expression may be a potential biomarker for HCC metastasis and poor patient survival.

β-arrestin1 and β-arrestin2, the main members of the arrestin family, are expressed ubiquitously. We also measured β-arrestin1 expression in a DEN-induced liver tumour model and HCC patients. Intriguingly, the results showed that β-arrestin1 expression did not change significantly during hepatocarcinogenesis and that β-arrestin1 expression also did not vary significantly among HCC patients with tumours of different histological grades (well-differentiated, moderately differentiated, or poorly differentiated) and normal controls. Additional *in vitro* studies showed that no significant differences in β-arrestin1 expression were observed among HCC cell lines with different metastatic potential and normal liver L-02 cells. Structural and functional differences between β-arrestin1 and β-arrestin2 may account for this divergence^37^. Although the structures of β-arrestin1 and β-arrestin2 are highly homologous, the N-terminal domain and the conformation of β-arrestin1 are different from those of activated β-arrestin2^38^,^39^.

Metastasis has always been a bottleneck with respect to tumour prognosis and therapy. Metastasis, both intrahepatic and extrahepatic, is particularly concerning and occurs in more than half of HCC cases. EMT, which is characterized by epithelial marker (E-cadherin) down-regulation or loss and mesenchymal marker (vimentin) up-regulation, is a crucial step in tumour invasion and metastasis. In a broad spectrum of cancers, including HCC^17^, invasion and metastasis progression may also involve localized EMT occurrences^40^, during which “abnormal epithelial cells” gradually display mixed epithelial and mesenchymal cell signatures characterized by loss of or decreases in cell-cell adhesion and polarity, as well as decreases in intercellular interactions, to rapidly transition into an aggressive phenotype to adapt microenvironments and ultimately establish secondary tumour lesions^41^. E-cadherin is a cell-cell adhesion protein that is thought to be a tumour suppressor and the primal factor governing EMT because it is silenced in many malignancies. In HCC patients, E-cadherin expression loss is correlated with a poor prognosis. Moreover, reduced E-cadherin expression is significantly associated with intrahepatic metastasis^42^. Vimentin has been recognized as a very important EMT marker, and its overexpression has been strongly associated with metastatic phenotypes and poor prognoses^43^. Here, we found that elevated E-cadherin and decreased vimentin expression are associated with β-arrestin2 overexpression. These results may have important implications regarding the role of β-arrestin2 in tumour progression, as they indicate that β-arrestin2 up-regulates E-cadherin expression and down-regulates vimentin expression, thereby participating in EMT.

The ERK pathway is activated in many types of human cancer and is recognized as a driving force for tumour initiation and progression^19^. Previous research also indicates that the PI3K/Akt pathway is involved in the pathogenesis of several human tumours, including HCC. The Akt and ERK signalling pathways were recently reported to play a key role in cancer EMT. Hepatitis B virus X protein represses miRNA-148a to enhance tumourigenesis by mediating HCC EMT through the Akt and ERK signalling pathways^44^. ERK/Akt also regulates EZH2 and E-cadherin to influence EMT in cancer^45^. TAAC3 promotes EMT through PI3K/Akt and ERK signalling pathway activation^46^. Hong KO and colleagues found that EMT can be reversed, as Akt inhibition restores E-cadherin expression and slows EMT in oral squamous cell carcinoma^47^. However, the relationships between β-arrestin2-mediated HCC metastasis and invasion inhibition and Akt and ERK1/2 signalling have not been elucidated. Therefore, we examined the changes in Akt and ERK1/2 activation in HCCLM3 cells and SMMC-7721 cells overexpressing β-arrestin2. Our results demonstrated that when we enhanced β-arrestin2 expression in HCC cells, E-cadherin expression increased, and vimentin expression and Akt activation decreased. By contrast, ERK phosphorylation did not change significantly. Our finding that β-arrestin2 mediates metastasis and invasion inhibition by altering E-cadherin, vimentin and p-Akt expression raised the possibility that β-arrestin2 prohibits Akt signalling activation and suppresses EMT, thereby reducing HCC cell invasion and metastasis.

In summary, we have provided evidence that β-arrestin2 is down-regulated in HCC and that β-arrestin2 overexpression impedes HCC migration and metastasis through Akt pathway inhibition. Our results show that β-arrestin2 plays an important role in HCC progression and metastasis. Future studies regarding the relationship between β-arrestin2 dysregulation and HCC metastasis may improve our understanding of the complicated mechanisms underlying HCC progression and may also facilitate the development of novel strategies for treating advanced HCC.

## Methods

### Mice and liver tumourigenesis

All mouse experiments were performed in accordance with the guidelines of the Animal Care and Use Committee of Anhui Medical University and were approved by the Ethics Review Committee for Animal Experimentation of the Institute of Clinical Pharmacology, Anhui Medical University. C57BL/6J mice were obtained from the Animal Center of Anhui Medical University. These mice were housed in a pathogen-free animal facility under a standard 12-h light/12-h dark cycle and provided *ad libitum* water and chow.

The DEN-induced mouse liver tumour model was established as described previously^48^. For long-term studies of liver tumour development and survival, mice at postnatal day 14 were administered single intraperitoneal injections of the genotoxic hepatocarcinogen DEN (Sigma-Aldrich, St. Louis, MO), which was dissolved in saline at a dose of 20 mg/kg body weight, and then weaned and maintained on regular chow. Littermates used as controls were injected with equal volumes of saline. The mice were sacrificed at 8, 16, 24, 32, and 40 weeks after DEN injection. Immediately after euthanasia, their livers were excised and photographed. These liver specimens were snap-frozen in liquid nitrogen for protein isolation or rapidly fixed in buffered 10% formalin for 16 h for histologic analysis.

### Patients and clinical specimens

Tumour specimens used for immunohistochemical staining were obtained from 75 HCC patients who underwent surgery for tumour resection between 2005 and 2008 in the Affiliated Hospital of Anhui Medical University. The clinicopathologic characteristics of these 75 patients are summarized in Table 1. Metastasis defined as intrahepatic metastasis and hepatic hilar lymph node metastasis. Normal liver samples were collected from 18 patients with intrahepatic biliary lithiasis. Nineteen samples each of tumour tissue and adjacent noncancerous liver parenchyma used for qRT-PCR assay and Western blot analysis were randomly collected from HCC patients who underwent surgical resection at the Affiliated Hospital of Anhui Medical University, Hefei, China. All tissues were collected immediately upon tumour resection in the operating theatre, snap-frozen in liquid nitrogen and then stored at −80 °C until use. This study was approved by the research ethics committee of Anhui Medical University, and all patients provided written informed consent to participate. The study was performed in accordance with the guidelines established by the Science Council of China.

### Cell lines

HCC cell lines with stepwise metastatic potential (MHCC97L, MHCC97H, and HCCLM3, which are hepatitis B virus [HBV]-positive cell lines with the same genetic background but different lung metastatic potentials)^49^, were purchased from Liver Cancer Institute, Zhongshan Hospital, Fudan University, Shanghai, China. The normal liver cell line L-02 and the HCC cell lines with relatively low metastatic potential HepG2 and SMMC-7721 were obtained from the Institute of Biochemistry and Cell Biology, Chinese Academy of Sciences, Shanghai, China. These 3 cell lines were HBV negative. All cell lines were maintained in high glucose DMEM (Life Technologies Inc., USA), supplemented with 10% FCS (Hyclone, Logan, UT), 100 IU/mL penicillin and 100 mg/mL streptomycin in humidified atmosphere of 5% CO_2_ at 37 °C.

### Histology and immunostaining

Formalin-fixed, paraffin-embedded liver tissues were sectioned at a thickness of 4 μm. For histological evaluation, H&E staining was performed using standard protocols. For immunohistochemical staining, tissue sections were deparaffinized in xylene and rehydrated using a graded series of ethanol/water solutions. These sections were retrieved in 10 mmol/L sodium citrate buffer (pH 6.0) in a microwave oven (300 W) for 10 min at 100 °C and then cooled to room temperature before being stained. Endogenous peroxidase activity was quenched by treatment with 3% H_2_O_2_ in methanol for 10 min. The sections were blocked in 0.25% normal goat serum in TBS for 1 h at room temperature and then incubated with primary antibodies to β-arrestin1 and β-arrestin2 (Santa Cruz Biotechnology, Santa Cruz, CA) for 1 h at 37 °C. Immunoreactivity was visualized using the streptavidin/peroxidase (SP) method (Zhongshan Goldenbridge Biotechnology Co., LTD, Beijing, China), according to the manufacturer’s protocol, and diaminobenzidine (DAB) was used as the chromogen. The nuclei were lightly counterstained with haematoxylin solution. A negative control was prepared using the same staining procedure but was not incubated with the abovementioned primary antibodies. Images were obtained using an Olympus BX53 microscope, and semiquantitative analysis was conducted using Image-Pro Plus software. Five random fields were analysed per slide, and the relative intensities of β-arrestin1 and β-arrestin2 expression were reflected by optical density values. To determine the correlation between β-arrestin2 expression and HCC patient clinicopathologic characteristics, β-arrestin2 protein staining intensity was scored semiquantitatively using a Quick-score (Q-score) method based on intensity and heterogeneity^50^,^51^. Staining intensity was scored as 0 (negative), 1 (weak), 2 (moderate), or 3 (strong). For heterogeneity scoring, the percentages of positive cells were divided into the following 4 grades: 0 (<10%), 1 (10–33%), 2 (34–65%), and 3 (66–100%). The Q-score of a given tissue sample was the sum of its intensity and heterogeneity scores and ranged from 0 to 6. β-arrestin2 positivity was determined using the following criteria:-(Q-score <1), + (Q-score = 1–2), ++ (Q-score = 3–4), and +++ (Q-score = 5–6). Immunohistochemical scoring was performed by 3 independent pathologists without knowledge of the patient characteristics.

### Immunofluorescence

Cells were plated on poly-D-lysine-coated coverslips in a 6-well dish. After 24 h, the cells were rinsed 3 times with PBS and fixed with 4% paraformaldehyde in PBS at room temperature for 20 min. The cells were then rinsed 3 times with PBS, permeabilized with 0.1% Triton X-100/PBS for 5 min at room temperature, and then blocked with 1% bovine serum albumin in PBS for 1 h at room temperature. The samples were then incubated with primary antibodies to β-arrestin2 (1:100 dilution) for 1 h before being washed with PBS, incubated with Alexa Fluor 555-conjuated mouse secondary antibodies for 1 h at room temperature, washed again with PBS, and mounted with Vectashield. Immunofluorescence images were obtained using a Leica laser scanning confocal microscope (TCS SP5).

### DNA transfection and siRNA transfection

For transient wild-type β-arrestin2 expression, we used a pcDNA3 expression plasmid encoding HA-β-arrestin2, which was kindly provided by Dr. Pei G. (Shanghai Institute for Biological Sciences, China). For transfection in six-well plates, HCCLM3 cells and SMMC-7721 cells were transiently transfected with the above β-arrestin2 vector using Lipofectamine 2000, according to the manufacturer’s recommendations (Invitrogen Life Technologies, Carlsbad, CA). Five micrograms of DNA was used per well.

For β-arrestin2 knockdown, SMMC-7721 cells and HepG2 cells were transfected with siRNA duplexes (GenePharma Co, China) featuring the following sequences specifically targeting β-arrestin2 RNA: (sense) CCAACCUCAUUGAAUUUGATT and (antisense) UCAAAUUCAAUGAGGUUGGTT. A scrambled RNA duplex was used as a negative control. The cells were allowed to grow for 48 h after transfection before being harvested for migration and invasion assays. Cell pellets were made using control and β-arrestin2-transfected cells 48 h after transfection. siRNA-mediated target knockdown was confirmed by Western blotting.

### Western blot analysis

For Western blot analysis, total protein was prepared from the HCC tissue samples and cell lines. Western blot was performed as described previously using several antibodies^52^. Briefly, liver tissue was homogenized in a homogenizing buffer containing 20 mM Tris-HCl (pH 7.4), 5 mM EDTA, 2 mM EGTA, 1.5 mM pepstatin, 2 mM leupeptin, 0.2 U/ml aprotinin, 0.5 mM phenylmethylsulfonyl fluoride, and 2 mM dithiothreitol, using a Polytron homogenizer. Cells were washed twice with ice-cold PBS and lysed in lysis buffer (0.5% NP-40, 50 mM Tris-HCl, pH 8.0, 100 mM NaCl, 1 mM phenylmethylsulfonyl fluoride (PMSF), 1 mM sodium orthovanadate, 10 μg/mL aprotinin and 10 μg/mL leupeptin) for 20 to 30 min on ice. Protein concentration was determined by the Bradford assay. The proteins were resolved by sodium dodecyl-sulfate-polyacrylamide gel electrophoresis (SDS-PAGE) and then transferred to polyvinylidene fluoride (PVDF) membranes (Millipore, Bedford, MA). The membranes were blocked with 5% non-fat dry milk in 0.05% Tween 20–PBS for 2 h and then incubated overnight at 4 °C with primary antibodies against β-arrestin1, β-arrestin2, E-cadherin (Santa Cruz Biotechnology, Santa Cruz, CA), vimentin, p-ERK1/2, ERK1/2, p-Akt, Akt (Cell Signaling, Danvers, MA), and β-actin. Immunoblot was done with the indicated primary antibody followed by the appropriate horseradish peroxidase (HRP)-conjugated secondary antibody for 2 h and visualized by ECL detection kit (Pierce Chemical, Rockford, IL). Autoradiographs were scanned using a Image-Pro Plus Imaging analysis software (Media Cybernetics, USA). All the experiments reported in this study were performed three times and the results were reproducible.

### Quantitative real-time PCR

Real-time PCR was carried out to determine β-arrestin1 and β-arrestin2 gene expression in tumour and nontumour liver tissue samples and HCC cell lines with stepwise metastatic potential. Total RNA was isolated using Trizol reagent (Invitrogen, Carlsbad, CA), and complementary DNA (cDNA) was synthesized using a RevertAid First Strand cDNA Synthesis Kit (Fermentas, Vilnius, Lithuania), according to the manufacturer’s instructions. Real-time PCR was carried out using a Real-time PCR Detection System (ABI 7500) using an SYBR GreenER qPCR SuperMix Universal Kit (Invitrogen, Carlsbad, CA), according to the manufacturer’s instructions. GAPDH cDNA amplification was used as an internal control for all real-time PCR amplification reactions. The primer sequences for each gene were as follows: β-arrestin1, forward primer: 5′-GGTAATAGATCTCCTTATCC-3′ and reverse primer: 5′-CCACAAGCGGAATTCTGTG-3′; β-arrestin2, forward primer: 5′-CCACGTCACCAACAATTCTG-3′ and reverse primer: 5′-TTGGTGTCTTCGTGCTTGAG-3′; and GAPDH, forward primer: 5′-TCAAGAAGGTGGTGAAGCAG-3′ and reverse primer: 5′-AGGTGGAAGAATGGGAGTTG-3′. The cycle threshold value was defined as the PCR cycle number at which the reporter fluorescence crossed the threshold. The cycle threshold value of each product was determined and normalized against that of the internal control, GAPDH.

### Wound-healing assay

Cells were seeded in six-well plates and incubated overnight in starvation medium. The cells were then transfected with the indicated pcDNA3/β-arrestin2-HA or β-arrestin2 siRNA for 48 h. The cell monolayers were subsequently wounded with a sterile 200 μL pipette tip and washed with starvation medium to remove detached cells. Wound closure was followed via microscopy at 24 h after wound infliction. The cells were photographed using an Olympus IX-71 inverted microscope. Wound healing was quantified as the mean percentage of the wound closure area relative to the area of the initial wound using Image J software.

### Invasion assay

Cell invasion was analysed using a modified Boyden chamber (Corning Costar, Rochester, NY, USA) containing a gelatin-coated polycarbonate membrane filter (6.5 mm diameter, 8 μm pore size). The upper surface of the filter was coated with 20 μL of Matrigel (BD Biosciences, Bedford, MA, USA). The cells were transfected with the indicated pcDNA3/β-arrestin2-HA or β-arrestin2 siRNA for 48 h. Then, the cells were trypsinized, 5 × 10^4^ cells in culture medium supplemented with 1% FBS were added to the upper chamber, and medium containing 10% FBS as a chemoattractant was added to the lower chamber. The cells were then incubated at 37 °C in 5% CO_2_. Noninvading cells were subsequently removed from the upper surface via scrubbing with a cotton swab, whereas cells that migrated to the lower surface of the filter were fixed with 3.7% (w/v) paraformaldehyde, stained with 0.5% (w/v) crystal violet, and counted using a light microscope.

### Statistical analysis

Statistical analyses were performed using SPSS software version 15.0 (SPSS, Chicago, IL). Cumulative survival time was calculated by the Kaplan-Meier method and analyzed by the log-rank test. Univariate and multivariate analyses were based on the Cox proportional hazards regression model. Values in figures are given as means and standard deviation of the mean if not otherwise indicated. The analysis of variance (ANOVA) and Student’s t-test are used in the SPSS software to determine significant differences between groups. Values of *P* less than 0.05 were considered to be significant.

## Additional Information

**How to cite this article**: Sun, W.-Y. *et al.* Down-regulation of β-arrestin2 promotes tumour invasion and indicates poor prognosis of hepatocellular carcinoma. *Sci. Rep.* **6**, 35609; doi: 10.1038/srep35609 (2016).

## Figures and Tables

**Table 1:** Correlation between β-arrestin2 expression and clinicopathologic characteristics in 75 HCC patients.

Clinicopathologic features	Case	β-arrestin2 expression				χ^2^	*P*
		−	+	++	+++		
Age (years)						4.108	0.250
<50	37	11	11	7	8		
≥50	38	20	7	5	6		
Sex						0.191	0.979
Male	62	25	15	10	12		
Female	13	6	3	2	2		
Liver cirrhosis						2.087	0.555
Positive	60	24	15	11	10		
Negative	15	7	3	1	4		
HBsAg						0.880	0.830
Positive	57	22	14	10	11		
Negative	18	9	4	2	3		
AFP (ng/ml)						1.613	0.656
<400	39	15	10	5	9		
≥400	36	16	8	7	5		
Tumor differentiation						13.190	0.040
Well differentiation	20	3	6	4	7		
Moderate differentiation	27	11	6	6	4		
Poor differentiation	28	17	6	2	3		
TNM stage						14.020	0.003
I-II	33	9	5	9	10		
III-IV	42	22	13	3	4		
Metastasis						9.403	0.024
Yes	35	19	10	3	3		
No	40	12	8	9	11		
Tumor size (cm)						10.985	0.012
<5	36	10	7	8	11		
≥5	39	21	11	4	3		

AFP, α-fetoprotein; TNM, tumor-node-metastasis; HBsAg, hepatitis B surface antigen.
